# Mitochondria-Related *TFAM* and *POLG* Gene Variants and Associations with Tumor Characteristics and Patient Survival in Head and Neck Cancer

**DOI:** 10.3390/genes14020434

**Published:** 2023-02-08

**Authors:** Ieva Golubickaite, Rasa Ugenskiene, Agne Bartnykaite, Lina Poskiene, Aurelija Vegiene, Evaldas Padervinskis, Viktoras Rudzianskas, Elona Juozaityte

**Affiliations:** 1 Department of Genetics and Molecular Medicine, Lithuanian University of Health Sciences, 44307 Kaunas, Lithuania; 2 Institute of Oncology, Lithuanian University of Health Sciences, 44307 Kaunas, Lithuania; 3 Department of Pathological Anatomy, Lithuanian University of Health Sciences, 44307 Kaunas, Lithuania; 4 Department of Otorhinolaryngology, Lithuanian University of Health Sciences, 44307 Kaunas, Lithuania

**Keywords:** head and neck cancer, *POLG*, *TFAM*, SNP, outcome, survival

## Abstract

In 2020, 878,348 newly reported cases and 444,347 deaths related to head and neck cancer were reported. These numbers suggest that there is still a need for molecular biomarkers for the diagnosis and prognosis of the disease. In this study, we aimed to analyze mitochondria-related mitochondrial transcription factor A (*TFAM)* and DNA polymerase γ (*POLG)* single-nucleotide polymorphisms (SNPs) in the head and neck cancer patient group and evaluate associations between SNPs, disease characteristics, and patient outcomes. Genotyping was performed using TaqMan probes with Real-Time polymerase chain reaction. We found associations between *TFAM* gene SNPs rs11006129 and rs3900887 and patient survival status. We found that patients with the *TFAM* rs11006129 CC genotype and non-carriers of the T allele had longer survival times than those with the CT genotype or T-allele carriers. Additionally, patients with the *TFAM* rs3900887 A allele tended to have shorter survival times than non-carriers of the A allele. Our findings suggest that variants in the *TFAM* gene may play an important role in head and neck cancer patient survival and could be considered and further evaluated as prognostic biomarkers. However, due to the limited sample size (*n* = 115), further studies in larger and more diverse cohorts are needed to confirm these findings.

## 1. Introduction

Head and neck cancer is a cancer type that affects various locations in the upper aerodigestive epithelium, such as the lip and oral cavity, oropharynx, larynx, and pharynx. Almost all head and neck cancers form in the epithelium of airways and are squamous cell carcinoma [1]. Despite arising from one cell type, this cancer type is heterogeneous due to its occurrence in different anatomical locations. Based on Global Cancer Observatory (GLOBOCAN)-reported data, in 2020, head and neck cancers accounted for 878,348 newly reported cases and 444,347 deaths [2]. The annual incidence in the United States in 2022 was projected to be approximately 66,470 new patients and ~15,050 new deaths [3]. The most-reported risk factors include excessive smoking, alcohol use, and oncogenic virus, such as human papillomavirus (HPV), infection [4]. The risk was estimated to be ten times higher for cigarette smokers than non-smokers, and alcohol consumption was also proven to be a risk factor and to enhance the smoking effect [5,6]. In older patients, head and neck cancer is associated with smoking and alcohol use. The number of such patients has decreased due to the reduced usage of tobacco [7]. HPV-positive and HPV-negative oropharyngeal cancers are different in their molecular and clinical parameters. HPV status impacts head and neck cancer. HPV-positive cancer is usually associated with a better prognosis, as it responds to treatment (radiation or chemotherapy) better [1,8,9]. However, HPV-associated cancer is more common among younger patients reflecting HPV type 16-induced head and neck cancer after oral sex exposure [10,11]. This shows that there are still unknown molecular mechanisms underlying head and neck cancer. However, Epidermal Growth Factor Receptor (EGFR) has been studied extensively, and it is known that it is upregulated in most (up to 90%) head and neck squamous cell carcinoma patients and is associated with a poor prognosis [12]. Head and neck cancer treatment is generally complex and requires specialists from multiple fields for the best patient outcome [13]. Treatment includes different options and combinations, such as surgery, chemotherapy, radiotherapy, and EGFR inhibitors [8]. Despite all current knowledge and available treatment options, there is a great need for biomarkers that would help in the early diagnosis and prognosis of the course of the disease and the more accurate prediction of the outcome. Changes in genes at the molecular level could be used as such biomarkers for the more detailed subtyping of head and neck cancer.

Single-nucleotide polymorphisms (SNPs) are among the most studied biomarkers in various diseases and health conditions. However, nuclear genes coding for mitochondrial proteins are rarely considered critical elements in carcinogenesis. Mitochondria are essential organelles important for energy generation, apoptosis, and metabolism. Moreover, they may play a critical role in carcinogenesis [14]. Since the mitochondrial genome encodes only 13 proteins, 22 transfer RNAs (tRNAs), and 2 ribosomal RNAs (rRNAs), the nuclear genome encodes other mitochondrial proteins (more than 1500) [15,16]. For example, mitochondrial transcription factor A (TFAM) is a nuclear-encoded protein essential in transcription, replication, and DNA damage sensing and repair [17,18,19,20,21,22]. In a previous study, lower expression of *TFAM* was found to be correlated with a decreased amount of mitochondrial DNA (mtDNA) [17]. Additionally, *TFAM* SNPs were reported to be associated with prostate, colorectal, breast, uterine, ovary, and cervical cancer and *TFAM* expression alterations in lung and breast cancer [23,24,25,26,27,28,29,30]. Another protein—DNA polymerase γ (POLG)—is the polymerase active in mitochondria and encoded by the nuclear *POLG* gene [31]. Mutations in this polymerase are known to cause mtDNA depletion and, therefore, decreased oxidative phosphorylation (OXPHOS) [32]. *POLG* mutations are usually studied in mitochondrial diseases, including ataxia, progressive external ophthalmoplegia, mitochondrial epilepsy, Alpers’ syndrome, Leigh’s syndrome, Parkinsonism, and male infertility [33,34,35,36,37,38]. They are not the usual targets in cancers, although several *POLG* SNPs were studied in breast, cervical, and colorectal cancer studies [23,32,39,40]. However, there is still a lack of knowledge about mitochondria-related nuclear SNPs such as *TFAM* and *POLG* in cancers. There are no previous studies of the selected *TFAM* and *POLG* polymorphisms in head and neck cancer patients. Therefore, we focused on SNPs in these genes and analyzed their associations with various disease and tumor pathomorphological characteristics, such as the stage, tumor grade, size, spread to regional lymph nodes, metastasis, and survival status in this study.

## 2. Materials and Methods

### 2.1. Study Subjects

Our study included one hundred fifteen patients diagnosed with head and neck cancer. Patients were included in this study if they were adults (older than 18 years old), were diagnosed with head and neck cancer without prior diseases, and agreed to participate in this study. After their initial diagnosis, patients were informed about the study in detail and signed the consent form. Their blood samples were collected afterward and stored until DNA extraction. Data about the condition, including the patient’s clinical data and tumor characteristics, including gender, age at diagnosis, stage, survival status, tumor localization, grade, size, regional lymph node status, and metastasis status were collected from medical records. To ensure confidentiality, all samples were depersonalized and assigned identification codes.

### 2.2. Genotyping

Genotyping was performed at the Institute of Oncology, Lithuanian University of Health Sciences (LUHS). DNA was purified from blood samples using the GeneJet Genomic DNA purification kit (Thermo Fisher Scientific, Waltham, MA, USA, Cat. K0721) according to the user’s manual. Selected single-nucleotide variants (rs11006132, rs11006129, rs1937, rs16912174, rs1692202, and rs3900887 in the *TFAM* gene and rs3087374, rs2072267, rs976072, and rs2307441 in the *POLG* gene) were determined with predesigned and commercially available TaqMan probe SNP Genotyping Assays (Thermo Fisher Scientific, Cat. #4351379) on a QuantStudio 3 Real-Time polymerase chain reaction (PCR) System (Thermo Fisher Scientific, Cat. #A28137). The standard genotyping PCR program was used: 95 °C for 10 min (polymerase activation), 95 °C for 15 s (denaturation), and 60 °C for 1 min (annealing and extension). The full protocol included 45 cycles of denaturation, annealing, and extension. Reactions were assembled into a 12 μL total volume and included 15 ng of DNA, 6.125 μL of TaqMan Universal Master Mix (Thermo Fisher Scientific, Waltham, MA, USA, Cat. #4304437), 0.625 μL of TaqMan SNP Genotyping Assay, and nuclease-free water. A no-template control (nuclease-free water) was used to confirm the lack of contamination in every run. Genotyping results were analyzed using an allelic discrimination plot, and genotypes were determined according to VIC and FAM fluorescence intensity after the run. The no-template controls were seen at the bottom left corner of the allelic discrimination plot for all runs, which indicated that there was no contamination.

### 2.3. Statistical Analysis

SPSS software version 28 (IBM Corp., Armonk, NY, USA) was used for statistical analysis. Associations between genotypes and disease or tumor parameters were evaluated with Pearson’s Chi-square or Fisher’s Exact tests and binary logistic regression with univariate and multivariate models (including various parameters). Survival analyses were performed using Kaplan-Meier analysis, and the difference between survival curves was analyzed using a log-rank test. Cox regression analysis was performed with univariate and multivariate models to check the impacts of additional factors (age at the time of diagnosis, tumor size T, status of regional lymph node involvement, and tumor grade) on survival. *p* < 0.05 was considered to indicate a statistically significant difference.

## 3. Results

### 3.1. Study Cohort Characteristics

This study included one hundred fifteen patients diagnosed with head and neck cancer. Most patients were males (*n* = 106), with only a fraction of females (*n* = 9). The median age in this cohort was 62 years (age range, 31–85 years). Larynx carcinoma was the most frequent head and neck cancer type among our patient group and had an 80.9% incidence rate, while the least frequent was oral cavity cancer, with only a 2.6% incidence rate (Table 1). Most patients were diagnosed with stage IV cancer (37.4%), had no metastasis (96.5%) or regional lymph node involvement (60.9%) reported, and were alive (61.7%) during the last censoring. Tumors were mostly grade G2 (75.7%), and their sizes varied from T1 to T4 in approximate distribution; the data are provided in Table 2.

### 3.2. TFAM and POLG Genotype and Allele Frequencies

We determined that *TFAM* rs11006132, rs11006129, rs1937, and rs16912174 and *POLG* rs3087374, rs2072267, rs976072, and rs2307441 were in Hardy-Weinberg equilibrium (HWE), and the distributions of all genotypes are presented in Table 3. Due to an amplification failure, four data points are missing from the *TFAM* rs3900887 genotyping data.

### 3.3. Association Analyses

Associations between polymorphisms and clinical or tumor characteristics data were evaluated using Pearson’s Chi-square test (Table 4 and Table 5). *TFAM* rs16912202 was excluded from further investigations because the TT genotype was the only genotype in our study group.

We found that *TFAM* rs3900887 and rs11006129 were significantly associated with the metastasis status (rs3900887 *p* = 0.007; rs11006129 *p* = 0.036) and patient survival status (rs3900887 *p* = 0.018; rs11006129 *p* = 0.019). Additionally, rs11006129 was associated with the tumor differentiation grade (*p* = 0.036), and rs3087374 in the *POLG* gene was associated with tumor size (*p* = 0.017). To further investigate significant associations, we grouped tumor size and differentiation grade data into subgroups; however, no statistically significant associations remained (Table 4 and Table 5). We removed metastasis status from further analysis due to the small count of metastasis (3.5%) events in our study group. No associations were found between *TFAM* rs11006132, rs1937, or rs16912174 or *POLG* rs2072267, rs976072, or rs2307441 polymorphisms and clinical data or tumor characteristics.

We continued investigating the significant associations between *TFAM* rs3900887 and rs11006129 and survival status; therefore, we performed logistic regression analyses. We found that patients with the *TFAM* rs3900887 AT genotype had an approximately 4.6 times higher death probability (odds ratio (OR) = 4.565; 95% CI, 1.554–13.414; *p* = 0.006) than those with the TT genotype.

Additionally, patients with the *TFAM* rs11006129 CT genotype had an almost 3 times higher death probability (odds ratio (OR) = 2.875; 95% CI, 1.166–7.086; *p* = 0.022) than those with the CC genotype. These associations also remained significant in multivariate genotype and allelic model analyses, where additional cofactors were included and are presented in Table 6.

### 3.4. Survival Analyses

**Figure 1:**
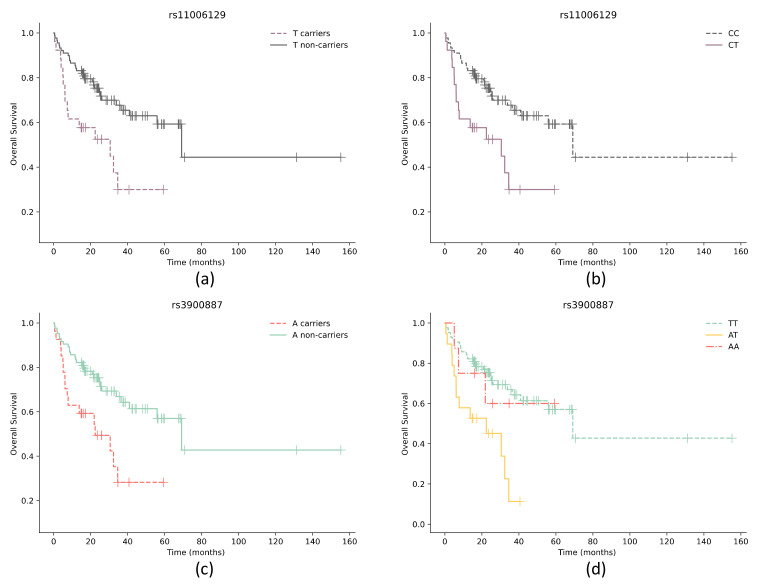
Overall survival of patients with different *TFAM* and *POLG* genotypes. Kaplan–Meier survival curves for overall survival of (**a**) *TFAM* rs11006129 T-allele carriers (*p* = 0.003); (**b**) patients with *TFAM* rs11006129 CC and CT genotypes (*p* = 0.003); (**c**) *TFAM* rs3900887 A-allele carriers (*p* = 0.011); (**d**) patients with *TFAM* rs3900887 AT and TT genotypes (*p* = 0.004) and TT and AT genotypes (*p* = 0.001). Censored cases are shown as a vertical line. The shadow with the same color represents the 95% confidence interval.

We analyzed all genotypes and alleles in our study to determine associations with overall patient survival. Kaplan–Meier plots for all significant associations are represented in Figure 1. Our findings suggest that both the *TFAM* rs11006129 T allele (*p* = 0.003) and CT vs. CC (*p* = 0.003) genotype are statistically significant for patient survival (Figure 1). Non-carriers of the *TFAM* rs11006129 T allele and those with the CC genotype survived significantly longer than T-allele carriers. This was followed by Cox univariate and multivariate regression analyses that included additional cofactors. In the Cox univariate analysis adjusted for age at diagnosis, it was determined that *TFAM* rs11006129 T-allele carriers had an approximately 2.5 times higher probability of shorter survival than non-carriers (hazard ratio (HR) = 2.505; 95% CI 1.331–4.713; *p* = 0.004). The differences remained significant in Cox multivariate analysis, following adjustment for other cofactors (tumor size T, regional lymph node status N, tumor grade G) (Table 7).

Additionally, we discovered that the *TFAM* rs3900887 A allele (*p* = 0.004) and AT vs. TT (*p* = 0.001) genotype are important for patient survival (Figure 1). We found that A-allele carriers had significantly shorter survival than non-carriers. Therefore, we expected that homozygous AA genotype patients would have the lowest survival rates; however, this was not the case. We hypothesized that this may possibly be due to the small sample size of AA genotypes (*n* = 8) and low death rates within this genotype (*n* = 3). In Cox univariate analysis adjusted for age at diagnosis, it was determined that A-allele carriers had an approximately two and a half times higher probability of shorter survival than non-carriers (hazard ratio (HR) = 2.460; 95% CI 1.319–4.588; *p* = 0.005). The differences remained significant in the Cox multivariate analysis following adjustment for other cofactors (tumor size T, regional lymph node status N, tumor grade G) (Table 7).

## 4. Discussion

Head and neck cancer affects various locations, such as the oral cavity, oropharynx, larynx, and pharynx. Most head and neck cancers are squamous cell carcinoma and form in the epithelium [1]. The major risk factors are excessive tobacco and alcohol consumption; however, HPV was also recognized as one of the risk factors and was associated with a better overall prognosis [4,41]. Despite the current knowledge about this cancer type, there are still 878,348 newly reported cases and 444,347 deaths worldwide each year [2], and multidisciplinary teams are required to achieve the best outcome and ensure the quality of a patient’s life.

Single-nucleotide polymorphisms, also known as SNPs, are among the widely studied biomarkers; however, mitochondrial DNA or nuclear DNA coding for mitochondrial proteins is rarely considered in cancer research. However, mitochondria are involved in major cellular processes and are considered to be important in carcinogenesis [14]. Since the nuclear genome encodes most of the mitochondrial proteins, we chose to analyze two of them. The first gene we selected was *TFAM*, which encodes mitochondrial transcription factor A. It is involved in crucial cellular processes, such as transcription, translation, replication, damage sensing, and mtDNA repair [17,18,19,20,21,22]. Additionally, the levels of mtDNA transcripts depend on the copy number of mtDNA, which is important for maintaining adenosine triphosphate (ATP) production [19,42]. The second gene we chose for analysis was *POLG*, which encodes the α subunit of polymerase γ, the only functioning polymerase in the mitochondria [31]. It is responsible for mtDNA replication, and *POLG* mutations are a known cause of mtDNA depletion and decreasedOXPHOS [32]. Mutations in *POLG* are often found in mitochondrial diseases, such as ataxia, progressive external ophthalmoplegia, mitochondrial epilepsy, Alpers’ syndrome, Leigh’s syndrome, Parkinsonism, and male infertility [33,34,35,36,37,38].

In this study, we analyzed rs11006132, rs11006129, rs1937, rs16912174, rs1692202, and rs3900887 in the *TFAM* gene and rs3087374, rs2072267, rs976072, and rs2307441 in the *POLG* gene. We selected these SNPs to be in both coding and non-coding sequences of each gene, to be benign in the general population, and to have a minor allele frequency (MAF) lower than 0.50 in the European population. Additionally, to the best of our knowledge, they were not previously studied in head and neck cancer. We found that rs11006129 and rs3900887 in the *TFAM* gene were significantly associated with patient survival. The rs11006129 variant is in the intron of the *TFAM* gene. The total MAF of this polymorphism in the 1000 Genome Project data was 0.15. However, the pathogenicity of this variant remains unclear, as it is predicted to be benign in Varsome [43] but is not reported in ClinVar. The *TFAM* rs11006129 CC genotype and non-carriers of the T allele had a probability of longer survival compared with those with the CT genotype or T-allele carriers. In our previous breast cancer patient cohort study, we found that patients with the rs11006129 T allele were less likely to have estrogen-positive tumors, and carriers of the C allele were less likely to develop tumors with vascular invasion [23]. Therefore, this confirms that the T allele may be a pathogenic factor for breast and head and neck cancer patients. However, no associations between rs11006129 and clinical data were found in the cervical cancer group [27]. The rs3900887 variant is in the intron of the *TFAM* gene. The total MAF of this polymorphism in the 1000 Genome Project data was 0.15. It is predicted to be benign by Varsome [43], while ClinVar lacks information on this variant, and the clinical significance is still unknown. We found that *TFAM* rs3900887 A-allele carriers tended to have shorter survival times than non-carriers. However, in the genotype analysis, we found that heterozygous patients with the AT genotype had the shortest survival times compared to both homozygous variants (TT or AA). From the results of the allele model, we expected AA homozygous patients to have the shortest survival. However, we believe that the small sample size of AA genotypes, with only eight patients and only three death events within this group, may have impacted the association. We analyzed rs3900887 in our previous cervical and breast cancer patient cohorts and found some statistically significant associations with tumor characteristics. For example, in cervical cancer, patients with *TFAM* rs3900887 TT and TA genotypes had a lower risk of larger tumors than patients with the AA genotype [27]. In the breast cancer group, we found that patients with the rs3900887 TT or TA genotype were associated with an increased risk of positive regional lymph nodes. Carriers of the TT genotype had an increased risk of estrogen receptor positivity or lymphatic invasion compared with patients with the AA genotype [23]. This SNP was previously analyzed in Alzheimer’s disease; however, no associations were found [44].

No associations were observed between disease characteristics and *TFAM* rs1937, rs11006132, or rs16912174. These variants are a missense variant (MAF 0.10), a non-coding transcript variant (MAF 0.24), and an upstream variant (MAF 0.01), respectively. One SNP, rs1692202, which is a non-coding transcript variant, MAF 0.12, was removed from statistical analyses because it only presented with the TT genotype in our study group. We did not find any previous studies focusing on these SNPs in head and neck cancer patients and evaluating SNP associations with disease characteristics. No significant associations were found with *POLG* rs2072267, rs3087374, rs976072, or rs2307441. *POLG* rs2072267 is an intron variant, MAF 0.47; rs3087374 is a missense variant, MAF 0.08; rs976072 is a downstream variant, MAF 0.39; and rs2307441 is a missense variant, 0.04. We also analyzed *POLG* rs2072267 in our previous cervical cancer study and determined that patients with the AA genotype survived longer without metastasis than those with the GG genotype [40]. We also found it to be associated with progression in breast cancer patients [23]. However, *POLG* rs2072267 was also investigated in Parkinson’s disease, ataxia, and colorectal cancer, but no significant results were reported. Our previous cervical cancer study found that the rs3087374 CA genotype was associated with the stage and tumor size compared to the CC genotype. Still, no associations were found in breast cancer or Parkinson’s disease studies [23,40,45]. Additionally, analyses of rs976072 in our previous breast and cervical cancer studies did not show any associations [23,40]. No significant results were reported in a study on bladder cancer [46]. Despite that, it was important for pancreatic cancer [47]. Similarly, in our previous breast cancer patient study, we found that rs2307441 was associated with tumor vascular invasion and metastasis-free survival [23]. However, our previous cervical cancer group study determined no associations between rs2307441 and clinical parameters, and no associations were reported in Parkinson’s or colorectal cancer [39,40,45].

There were limiting factors in this study, including a limited number of patients, as there were only one hundred fifteen patients in our cohort, and only nine of them were females. Additionally, only ten SNPs were selected and analyzed in *TFAM* and *POLG* genes that were reported to be benign in the general population. Despite these limitations, our results suggest that mitochondrial transcription factor A, encoded by the nuclear *TFAM* gene, may play an important role in head and neck cancer patient survival and could potentially serve as a prognostic survival biomarker. Therefore, further studies in larger and more diverse cohorts are needed to confirm our findings. After confirming *TFAM* SNPs in larger cohorts, future directions could potentially include the construction of the mutation by CRISPR/Cas9 or ectopic expression analysis and the evaluation of their impact on protein expression and translational activity.

## Figures and Tables

**Table 1:** Frequency of head and neck cancer localization in our cohort.

Localization	Frequency
Larynx	93 (80.9%)
Pharynx	14 (12.2%)
Paranasal and nasal	5 (4.3%)
Oral cavity	3 (2.6%)

**Table 2:** The distribution of tumor and clinical characteristics.

Characteristics		Frequency
Grade	1	14 (12.2%)
	2	87 (75.7%)
	3	13 (11.3%)
	4	1 (0.9%)
Size	T1	36 (31.3%)
	T2	22 (19.1%)
	T3	27 (23.5%)
	T4	30 (26.1%)
Cancerous regional lymph nodes	N0	70 (60.9%)
	N1	45 (39.1%)
Metastasis	Absent	111 (96.5%)
	Present	4 (3.5%)
Stage	I	32 (27.8%)
	II	23 (20.0%)
	III	17 (14.8%)
	IV	43 (37.4%)
Death fact	Alive	71 (61.7%)
	Deceased	44 (38.3%)

**Table 3:** The distribution of *TFAM* and *POLG* genotype and allele frequencies.

Gene and SNP	Count	Genotype Frequency	Allele Frequency
*TFAM* rs11006132	56	AA—0.49 AG—0.47 GG—0.04	A—0.72 G—0.28
	54		
	5		
*TFAM* rs11006129	89	CC—0.77 CT—0.23	C—0.89 T—0.11
	26		
*TFAM* rs1937	83	GG—0.72 CG—0.27 CC—0.01	G—0.86 C—0.14
	31		
	1		
*TFAM* rs16912174	107	TT—0.93 GT—0.07	T—0.97 G—0.03
	8		
*TFAM* rs1692202 *	115	TT—1.00	T—1.00
*TFAM* rs3900887 *	84	TT—0.76 AT—0.17 AA—0.07	T—0.84 A—0.16
	19		
	8		
*POLG* rs3087374	9520	CC—0.83 AC—0.17	A—0.09 C—0.91
*POLG* rs2072267	12	AA—0.10 AG—0.54 GG—0.36	A—0.37 G—0.63
	62		
	41		
*POLG* rs976072	20	AA—0.17 AG—0.56 GG—0.27	A—0.45 G—0.55
	64		
	31		
*POLG* rs2307441	97	TT—0.84 CT—0.16	T—0.92 C—0.08
	18		

The table presents the genotype distribution within our study cohort. Gene and single-nucleotide polymorphism (SNP) rs numbers are provided. Patient count together with the genotype frequency is provided for each genotype within the cohort, together with the allele frequency for every polymorphism. * Genotype distribution is not in Hardy-Weinberg equilibrium.

**Table 4:** *TFAM* gene genotype associations with disease and tumor characteristics.

Variable	*p*-Value				
	rs3900887	rs11006132	rs11006129	rs1937	rs16912174
Stage (I, II, III, IV)	0.815	0.145	0.577	0.534	0.455
T (T1, T2, T3, T4)	0.443	0.149	0.824	0.634	0.480
T (T1, T2 and T3, T4 groups)	0.174	0.877	0.236	0.596	0.349
N (negative vs. positive)	0.751	0.998	0.437	0.627	0.596
M (negative vs. positive)	0.007	0.550	0.036	0.977	0.746
Differentiation grade G (G1, G2, G3, G4)	0.066	0.284	0.036	0.948	0.147
Differentiation grade G (G1, G2 vs. G3, G4)	0.486	0.132	0.341	0.694	0.658
Survival status	0.018	0.691	0.019	0.321	0.638

T—tumor size; N—regional lymph node status; M—metastasis status; G—tumor grade.

**Table 5:** *POLG* gene genotype associations with disease and tumor characteristics.

Variable	*p*-Value			
	rs3087374	rs2307441	rs2072267	rs976072
Stage (I, II, III, IV)	0.054	0.756	0.314	0.102
T (T1, T2, T3, T4)	0.017	0.788	0.494	0.934
T (T1, T2 and T3, T4 groups)	0.420	0.415	0.209	0.958
N (negative vs. positive)	0.199	0.401	0.336	0.996
M (negative vs. positive)	0.139	0.499	0.422	0.437
Differentiation grade G (G1, G2, G3, G4)	0.897	0.802	0.919	0.833
Differentiation grade G (G1, G2 vs. G3, G4)	0.455	0.314	0.873	0.585
Survival status	0.076	0.102	0.449	0.646

T—tumor size; N—regional lymph node status; M—metastasis status; G—tumor grade.

**Table 6:** Univariate and multivariate logistic regression analyses between *TFAM* rs3900887 and rs11006129 and fact of death.

Dependent	SNP	Covariates	Model No. 1				Model No. 2			
			OR	95% CI		*p*	OR	95% CI		*p*
Survival status	rs3900887	AT vs. TT	4.565	1.554	13.414	0.006	5.891	1.698	20.447	0.005
		Age *	0.989	0.981	0.996	0.002	0.942	0.915	0.971	0.001
		T					1.344	0.857	2.108	0.198
		N					3.041	1.031	8.965	0.044
		G (G1, G2 vs. G3, G4)					4.646	1.264	17.079	0.021
Survival status	rs3900887	A-allele carriers vs. non-carriers	2.940	1.198	7.214	0.019	3.613	1.274	10.250	0.016
		Age *	0.989	0.982	0.996	0.003	0.986	0.942	1.032	0.551
		T					1.651	1.050	2.596	0.030
		N					2.887	1.021	8.165	0.046
		G (G1, G2 vs. G3, G4)					6.589	1.593	27.262	0.009
Survival status	rs11006129	CT vs. CC	2.875	1.166	7.086	0.022	3.679	1.288	10.511	0.015
		Age *	0.989	0.982	0.996	0.001	0.986	0.943	1.032	0.546
		T					1.665	1.057	2.622	0.028
		N					2.928	1.045	8.205	0.041
		G (G1, G2 vs. G3, G4)					6.938	1.678	28.686	0.007
Survival status	rs11006129	T-allele carriers vs. non-carriers	2.875	1.166	7.086	0.022	3.930	1.362	11.341	0.011
		Age *	0.989	0.982	0.996	0.001	0.989	0.945	1.035	0.624
		T					1.619	1.037	2.528	0.034
		N					1.985	1.145	3.442	0.015
		G (G1, G2 vs. G3, G4)					6.997	1.679	29.162	0.008

* Age at the time of diagnosis; OR—odds ratio; CI—confidence interval; T—tumor size; N—regional lymph node status; G—tumor grade. Model No. 1—logistic regression analysis adjusted for age at diagnosis. Model No. 2—logistic regression analysis adjusted for age at diagnosis, tumor size, regional lymph node status, and tumor grade.

**Table 7:** Cox multivariate model with additional cofactors for patients’ overall survival.

Variables	HR	95% CI	*p*-Value
rs11006129			
T-allele carriers	2.982	1.543–5.765	0.001
Age *	0.999	0.965–1.035	0.958
T	1.704	1.239–2.342	0.001
N	1.604	0.794–3.239	0.188
G (G1, G2 vs. G3, G4)	2.743	1.291–5.831	0.009
rs3900887			
A-allele carriers	2.751	1.437–5.266	0.002
Age *	1.001	0.967–1.038	0.934
T	1.672	1.221–2.289	0.001
N	1.537	0.756–3.127	0.235
G (G1, G2 vs. G3, G4)	2.634	1.236–5.612	0.012

* Age at the time of diagnosis; HR—hazard ratio; CI—confidence interval; T—tumor size; N—regional lymph node status; G—tumor grade.

## Data Availability

The data presented in this study are available on request from the corresponding author.
